# Synthetic T-Cell Receptor-like Protein Behaves as a Janus Particle in Solution

**DOI:** 10.1021/jacs.4c08932

**Published:** 2024-12-19

**Authors:** Emily Sakamoto-Rablah, Jordan Bye, Arghya Modak, Andrew Hooker, Shahid Uddin, Jennifer J. McManus

**Affiliations:** † HH Wills Physics Laboratory, University of Bristol, Tyndall Avenue, Bristol BS8 1TL, U.K.; ‡ Immunocore Limited, 92 Milton Park, Abingdon OX14 4RY, U.K.; § Bristol Biodesign Institute, University of Bristol, Bristol BS8 1QU, U.K.

## Abstract

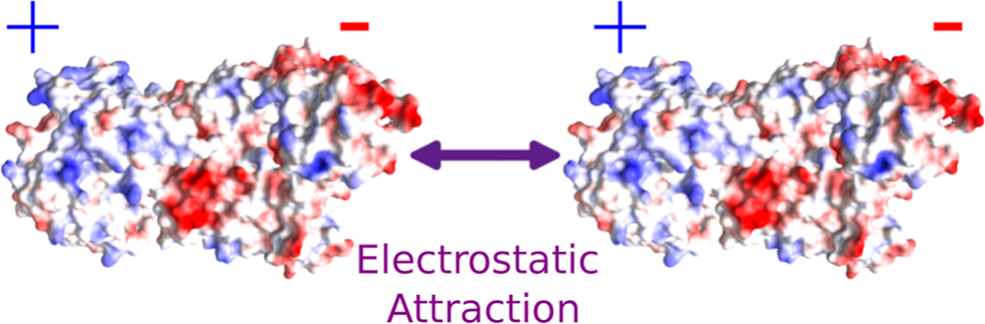

Protein engineering enables the creation of tailor-made proteins for a variety of applications. ImmTACs stand out as promising therapeutics for cancer and other treatments while also presenting unique challenges for stability, formulation, and delivery. We have shown that ImmTACs behave as Janus particles in solution, leading to self-association at low concentrations, even when the average protein–protein interactions suggest that the molecule should be stable. The formation of small but stable oligomers was confirmed by static and dynamic light scattering and analytical ultracentrifugation. Modeling of the structure using AlphaFold leads to a rational explanation for this behavior, consistent with the Janus particle assembly observed for inverse patchy particles.

## Introduction

Recent advances in protein engineering have allowed for tailor-made proteins with high selectivity to be developed as therapeutic products.^1^ This technological advancement holds promise for numerous novel applications, including the design of artificial organelles with custom functions, creating new biobased materials, or developing new pharmaceutical molecules. Among the latter, one such class of therapeutic molecules is the immune mobilizing monoclonal T-cell receptor (TCR) against cancer (ImmTAC).^2^ ImmTACs target cancerous cells with high specificity by design while harnessing the body’s own immune response and are unique molecules in the biopharmaceutical space.

ImmTAC molecules consist of an affinity enhanced TCR joined by a flexible linker to an anti-CD3 single-chain variable fragment (scFv).^3,4^ The TCR region binds to cancer cells with high affinity, while the scFv component mobilizes T-cells within the body. This innovative approach holds tremendous potential to deliver life-saving treatments to cancer patients; one ImmTAC molecule (tebentafusp) has FDA approval as a treatment for metastatic uveal melanoma, an aggressive form of eye cancer with previously limited treatment options.^5^ As research into ImmTACs (and other synthetic proteins) continues, more options for previously hard-to-treat diseases now seem tractable.

The ability to create proteins not found in nature has profound implications for the solution stability. Natural proteins have been finely tuned by evolution to be structurally and colloidally stable under physiological conditions, maintaining a delicate balance between solubility and aggregation to ensure proper cellular function. In contrast, synthetic proteins such as ImmTACs offer a unique opportunity to explore the effects of deliberate design modifications optimized for function and the consequences for solution stability and therefore formulation and delivery. These modifications, aimed at enhancing target specificity and immune activation, may also impact the protein phase behavior and self-assembly.

Understanding weak, nonspecific protein–protein interactions (PPIs) and self-assembly to select formulation conditions is central to realizing the transformative potential of these molecules. Ensuring colloidal and structural stability is a crucial aspect of producing safe and effective drug products.^6−8^ Colloidal stability is highly dependent on weak, nonspecific PPIs as well as interactions of the protein with solvent and cosolutes. Current manufacturers of biotherapeutics often rely on previously successful formulations or use expensive and time-consuming trial-and-error approaches due to the complex nature of protein intermolecular interactions which has consequences for their phase behavior. As more novel and innovative biotherapeutics such as ImmTACs are developed for which prior formulation strategies do not exist, established techniques for selecting solution conditions and excipients as routes to formulation need re-evaluation, and bringing them to market will require a shift to more rational approaches.

Here, we present the first study of the weak self-interactions of ImmTAC molecules. We demonstrate that the design of an ImmTAC molecule targeting the NY-ESO-1 antigen (referred to henceforth as ImmTAC1) for biologically optimized function produces a highly anisotropic surface charge distribution, resulting in the unexpected formation of noncovalent oligomers. We then compare ImmTAC1 with a second molecule (ImmTAC2) and demonstrate that a more extreme Janus-like nature leads to more dramatic self-association behavior. ImmTAC1 serves as a model protein representative of this class of potentially transformational molecules, highlighting the formulation challenges faced for this particular ImmTAC and other therapeutic bispecific fusion proteins.

## Results and Discussion

### ImmTAC Structure and Structural Stability

**Figure 1:**
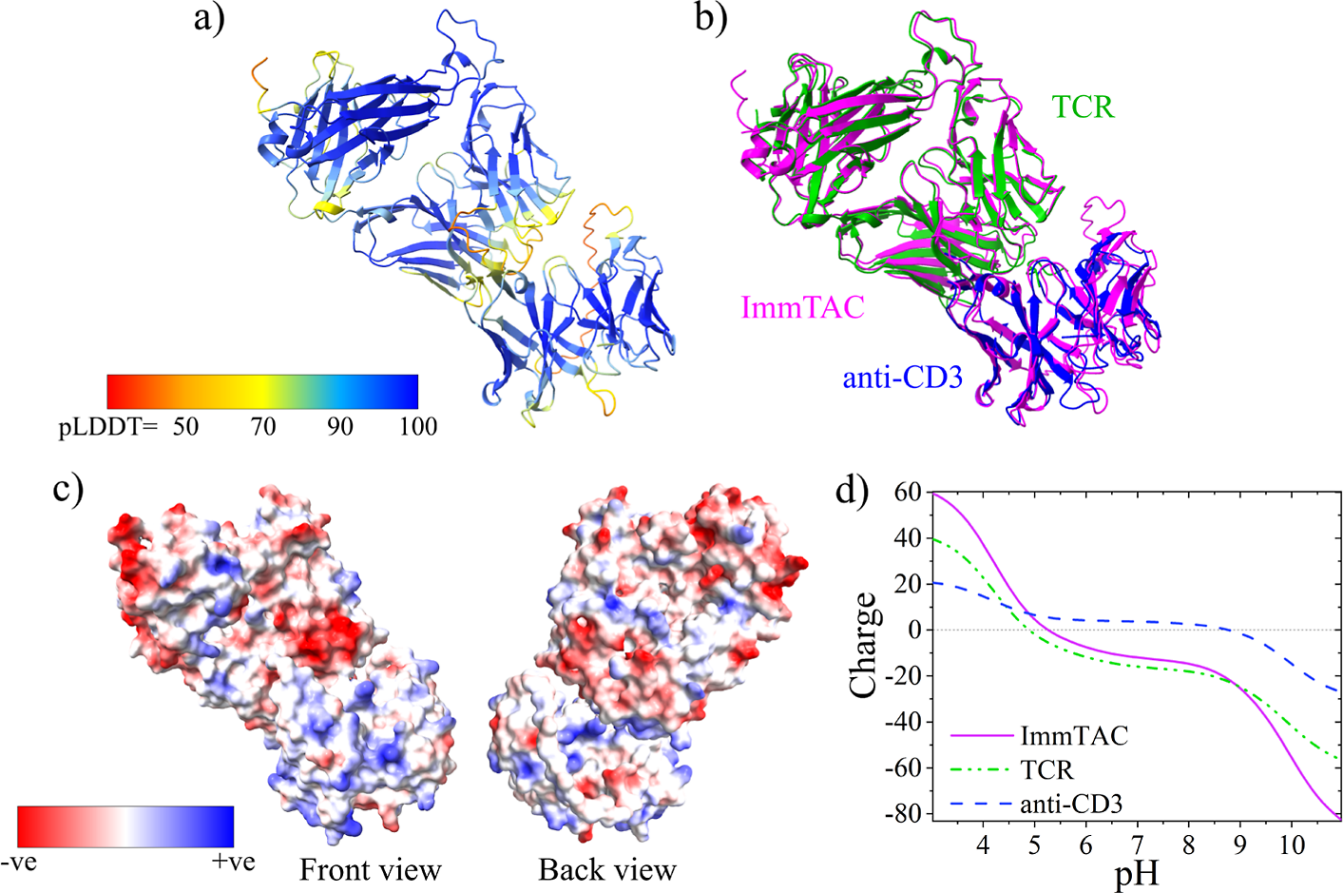
(a) Predicted structure of ImmTAC1 generated by AlphaFold, colored by confidence metric pLDDT. (b) AlphaFold structure (magenta) overlaid with the crystal structure of TCR (PDB 2P5E) (green) and anti-CD3 (PDB 1XIW) (blue). (c) Front and back view of the predicted ImmTAC surface colored by charge at pH 7.0. (d) Estimated total electrostatic charge on ImmTAC, TCR, and anti-CD3 regions.

While the structure of ImmTAC1 has not yet been successfully obtained by crystallography or cryo-EM methods, we can use existing crystal structures for the constituent domains (TCR and anti-CD3 scFv) previously reported in the literature, which we have combined with validation from structural predictions using AlphaFold.^9^ The structure of the TCR portion of ImmTAC1 has been determined by X-ray crystallography and reported previously (PDB: 2P5E). Structure 2P5E is the same TCR variant which was used as the basis for fusion protein ImmTAC1, evidenced by the fact that when compared in a sequence alignment, 2P5E and ImmTAC1 showed a 99.0% sequence identity across 195 residues in the α-chain and 100.0% across 241 residues in the β-chain. We analyzed this alongside an anti-CD3 antibody fragment reported in the literature (PDB: 1XIW). While structure 1XIW is not a perfect sequence match with the anti-CD3 section of ImmTAC1, it does have a high degree of sequence similarity, with 77.6% identity across 228 residues (when neglecting the flexible linker in the ImmTAC1 scFv section, which is not expected to have any secondary or tertiary structure). Sequence alignments were conducted using default settings in the “SIM” tool available on the ExPASy server.^10^ In order to ascertain which residues are situated on the protein’s surface, AlphaFold was employed alongside analyses of these structures. Based on the amino acid sequence of ImmTAC1, five predicted structures were generated by AlphaFold. Among these, the structure that ranked highest by the predicted local distance difference test (pLDDT) is shown in Figure 1a. The pLDDT is a per-residue confidence metric which estimates how well the prediction would match the real structure based on the local distance difference test.^11^ The highest-ranking structure had a total average pLDDT of 86.9%. As illustrated in Figure 1a, most lower pLDDT regions are those in which we expect structural disorder such as the linkers within the scFv portion and between the TCR and scFv, rather than areas with significant secondary structure. To further validate the AlphaFold prediction, Figure 1b shows the predicted structure overlaid with structures 2P5E and 1XIW. The predicted structure shows good spatial overlap with the experimentally determined structures, having a root mean squared deviation (RMSD) of 0.624 and 0.672 Å, respectively, between the AlphaFold structure and PDB structures.

Since we have validated the structure generated by AlphaFold by comparison with the experimentally determined structures, we may use the predicted model to analyze which charged residues sit at the protein’s surface and therefore contribute to electrostatic PPIs. The online Adaptive Poisson–Boltzmann Solver (APBS) tool was used to assign electrostatic charges to the AlphaFold structure at different pH values.^12^ A visualization of the resulting surface charge at pH 7.0 is shown in Figure 1c. The TCR section of the molecule carries a largely negative charge, while the anti-CD3 portion is positively charged based on surface-exposed charge densities. This is further illustrated by the net charge of the protein at different pH values (calculated using Prot-pi^13^) compared with that of the respective sections (i.e., the scFv and TCR), shown in Figure 1d. This is a feature of the way the protein has been designed and engineered in that creating a fusion of two proteins with contrasting isoelectric points results in a molecule with a net charge of −12.0 at pH 7 but an anisotropic distribution of that charge.

### Measurement of PPIs

**Figure 2:**
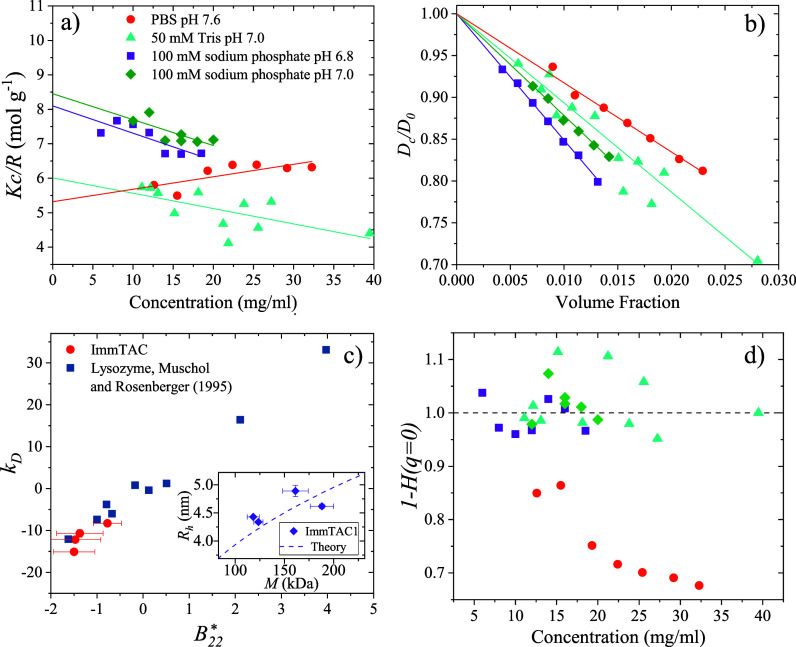
(a) SLS data plotted as a Debye plot. Straight lines are linear fits to data with slopes equal to the second virial coefficient *B*_22_. (b) Diffusion data measured by dynamic light scattering (DLS). Straight lines show fits to the data with the slope equal to interaction parameter *k*_D_. (c) Relationship between the interaction parameter and second virial coefficient for ImmTAC1 and lysozyme. Data for lysozyme is reproduced from Muschol and Rosenberger.^15^ Inset shows the relationship between the measured hydrodynamic radius and molecular weight for ImmTAC1 along with the expected relationship (dashed line) calculated from the theory (eq 17). (d) Hydrodynamic function calculated using eq 12 as a function of concentration.

To evaluate the strength of net PPIs, static light scattering (SLS) was carried out in 100 mM sodium phosphate at pH 6.8 and 7.0, in phosphate buffered saline (PBS) at pH 7.4, and in 50 mM Tris at pH 7. The SLS data are shown in Figure 2a in the form of a Debye plot. We fit a straight line according to the Debye–Zimm equation
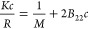
1where *c* is the protein concentration, *R* is the excess Rayleigh ratio (defined as the ratio of scattered light to incident light at a given angle, in this case, 90°), *M* is the protein molecular weight, and *K* is an instrument constant given by
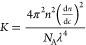
2with *n* equal to the sample refractive index,  the refractive index increment (here, taken to be 0.19 in accordance with Zhao et al.^14^), *N*_A_ Avogadro’s constant, and λ the laser light wavelength (632.8 nm). The second virial coefficient *B*_22_ is in units of volume moles per mass squared. *B*_22_ represents deviations to the osmotic pressure introduced by pairwise interactions and hence is often used as a measure of net interaction strength. This is often reported in units of volume, , and is defined as^15^
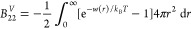
3where *r* is the protein–protein separation, *w*(*r*) is the average interprotein interaction potential, *k*_B_ is Boltzmann’s constant, and *T* is temperature in Kelvin. According to this definition, positive values of *B*_22_ correspond to net repulsion between protein molecules (and therefore conditions which are likely conducive to colloidal stability), while negative values reflect net attractive interactions and a likely tendency toward aggregation.^15^ All molecules experience interparticle repulsion at short range due to steric exclusion, which contributes a positive component to the value of *B*_22_. Using eq 3 and approximating the particles as hard spheres, this excluded volume contribution is *B*_22_^*V*,ex^ = 4*V*_HS_, where *V*_HS_ is the hard sphere volume, i.e., *B*_22_^*V*,ex^ is equal to the excluded volume per particle. Simulations have shown that *B*_22_^*V*,ex^ is well approximated by the excluded volume per particle of a sphere with the radius equal to the hydrodynamic radius *R*_h_.^16^ Here, we assume the relation between molecular weight *M* and radius of gyration *R*_g_ to be
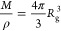
4where ρ is the protein density, taken here to be 1.4 g/cm^3^,^17^ while we use the molecular weight calculated based on the amino acid sequence of ImmTAC1 to be 77.2 kDa (calculated using the ProtParam tool available on the ExPASy server^18^). For globular proteins, the ratio of *R*_g_ and *R*_h_ is generally found to be 0.775;^19^ hence, we estimate
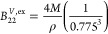
5

The net contributions of all other pair interactions (i.e., “soft” interactions) normalized by the hard sphere contributions are often represented as
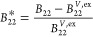
6known as the reduced second virial coefficient. Values obtained from fits to the data in Figure 2a are listed in Table 1. The fitted *B*_22_^*^ values obtained by SLS indicate net attractive interactions between protein molecules.

**Table 1:** Second Virial Coefficient and Molecular Weight Values Measured by SLS and *k*_D_ and Hydrodynamic Radius Values Measured by DLS

solution condition	*B*_22_^*^	molecular weight (kDa)	*k*_D_	hydrodynamic radius (nm)
PBS pH 7.6	–0.7 ± 0.3	190 ± 10	–8.3 ± 0.3	4.62 ± 0.04
50 mM tris pH 7.0	–1.4 ± 0.5	160 ± 10	–10.7 ± 0.9	4.9 ± 0.1
100 mM sodium phosphate pH 7.0	–1.5 ± 0.6	118 ± 6	–12.2 ± 0.4	4.42 ± 0.02
100 mM sodium phosphate pH 6.8	–1.5 ± 0.5	123 ± 5	–15.1 ± 0.3	4.34 ± 0.03

DLS was also used to measure the net interaction parameter *k*_D_ using the same solution conditions in order to validate the SLS results. The intensity correlation function is given by
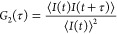
7where *I* is intensity of scattered light, *t* is time, and τ is a lag time, effectively comparing the intensity at a given time *I*(*t*) to that at a later time *I*(*t* + τ). For monodisperse solutions, the correlation function takes the form^20^
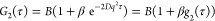
8where *D* is the diffusion coefficient and *q* is the Bragg wave vector, related to the refractive index *n* of the solvent, wavelength of scattered light λ, and the scattering angle θ by . *B* and β are the amplitude (or intercept) and baseline of the correlation function, respectively, while *g*_2_(τ) is referred to as the normalized correlation function. In a polydisperse solution^21^
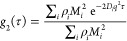
9where ρ is the number density and subscript *i* denotes each species in the solution. We may fit ln(*g*_2_(τ)) to a second-order polynomial
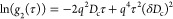
10where the first-order coefficient gives the collective diffusion coefficient, while the second-order coefficient corresponds to fluctuations in the diffusion coefficient as described by Roberts et al.^21^ From the collective diffusion coefficient, we may derive the net interaction parameter *k*_D_, using^15^
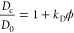
11where *D*_0_ is the diffusion coefficient of the protein extrapolated to infinite dilution, and ϕ = ν*c* is the protein volume fraction with partial specific volume ν of the protein taken here to be 0.73 mL/g. Shown in past studies to correlate with the second virial coefficient for some globular proteins,^21^ a positive *k*_D_ value is indicative of net repulsive interactions, while negative *k*_D_ indicates net attractive interactions.^22^ However, *k*_D_ differs from *B*_22_ in that there is a contribution from hydrodynamic, or indirect, interactions (since diffusion is affected by both hydrodynamics and thermodynamics), while *B*_22_ is a purely thermodynamic quantity. Collective diffusion coefficients for ImmTAC1 are listed in Figure 2b. Values of *k*_D_ obtained by a linear fit according to eq 11 are given in Table 1. In agreement with the *B*_22_ values, the fitted *k*_D_ values indicate net attractive PPIs. In both SLS and DLS plots, data taken at the lowest concentrations have been excluded because the polydispersity indices are relatively high at these concentrations and hydrodynamic radius values are highly variable, indicating potential aggregation. As such, data at the lowest concentrations were not included in the fitting of interaction parameters. Examples of such data at low concentrations are shown in Figure S12 of the Supporting Information.

In order to test the agreement between the data obtained from SLS and DLS, *k*_D_ values were plotted against *B*_22_, along with values for lysozyme, obtained by Muschol and Rosenberger^15^ and are shown in Figure 2c. Our data show good agreement with the linear relationship measured by Muschol and Rosenberger^15^ within errors (taken to be the error on the gradient given by the linear fits).

If we were to consider only the net charge of the protein, this result that interprotein interactions are measured to be net attractive in nature may appear to be somewhat surprising. The pH range studied here (6.8–7.4) is higher than the theoretical isoelectric point of 5.27, with theoretical values for net charge being between −9.8 and −13.5 (Figure 1d). Based on these values of net charge, we might expect double layer repulsion to dominate the net interaction potential at low ionic strengths. Attractive pairwise interaction potentials in charged proteins have been explained in past studies as an attractive electrostatic force between oppositely charged “patches” on the surface of molecules with anisotropic surface charge density.^21,23,24^ This is consistent with the asymmetry in surface charge observed from the AlphaFold models in Figure 1c and is evident in the contrast in charge between the TCR and scFv sections (Figure 1d). It is reasonable to suggest that this results in electrostatic attraction with the negatively charged TCR, and positively charged scFv of two different ImmTACs will experience a charge–charge attraction at sufficiently small intermolecular distances. Indeed, past simulations have also shown the importance of considering anisotropy to explain protein phase behavior.^21,25−27^

Since DLS measures contributions from hydrodynamic interactions, while SLS does not, data from these two techniques may be combined to ascertain the strength of hydrodynamic interactions. The hydrodynamic function *H* reflects how the drag force on a protein is affected by solvent flow due to surrounding proteins. It can be shown that^15^
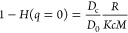
12

Resulting values for hydrodynamic functions are shown in Figure 2d. Values for *H* are seen to be distributed around 0 for all solution conditions other than for PBS at pH 7.6, i.e., hydrodynamic interactions only play a significant role in the protein interactions in PBS. According to the measured *B*_22_ and *k*_D_ values, the protein has the least attractive net PPIs in PBS compared with the other buffers examined. PBS contains a high proportion of NaCl, which provides an electrostatic screening. This implies that in the case of high screening, the direct interactions of electrostatic origin are suppressed sufficiently that hydrodynamic (indirect) interactions have a measurable effect, while under other solution conditions, the weak intermolecular electrostatic interactions dominate to the point of suppressing the hydrodynamic interactions. This sensitivity of the net interaction potential and solution behavior to ionic strength of the solution conditions further supports the hypothesis that electrostatics is the dominant interaction.

**Figure 3:**
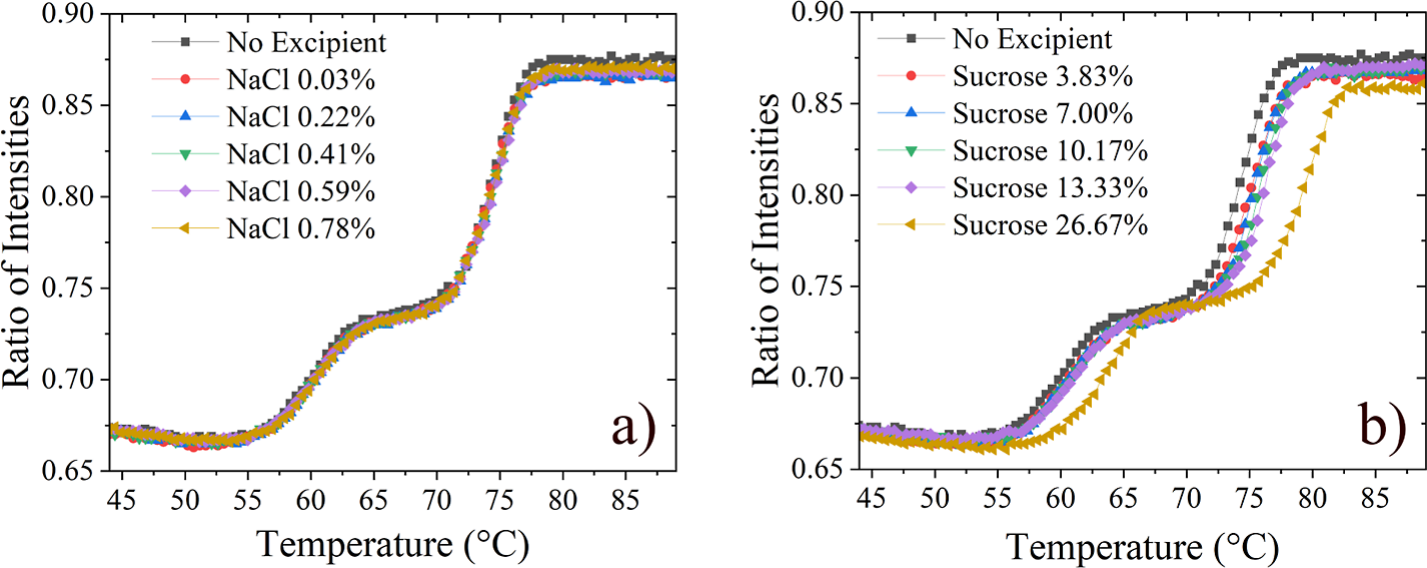
Thermal unfolding curves measured by nano-DSF in the presence of various concentrations of NaCl (a) and sucrose (b). Concentrations of NaCl and sucrose are in (w/v) percentage.

In order to rule out this difference being due to structural changes such as unfolding, nano differential scanning fluorimetry (nano-DSF) was used to measure structural changes with temperature in the presence of various cosolutes. The temperature of the samples was varied between 20 and 100 °C and the ratio of fluorescence intensities at 355 and 330 nm was measured in order to monitor the degree of unfolding. The temperature at which ImmTAC1 unfolds (*T*_m_) was measured in 100 mM sodium phosphate buffer at pH 7.0, shown in Figure 3. The data show two unfolding transitions, corresponding to the scFv and TCR section. Data were fitted to the following three-state, biphasic function
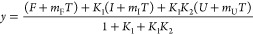
13
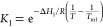
14
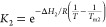
15where *y* is the ratio of intensities, *F*, *I*, and *U* are the intercepts of the lower, intermediate, and upper plateaus, respectively, *m*_*F*_, *m*_I_, and *m*_U_ are the gradients of the respective plateaus, Δ*H*_1_ and Δ*H*_2_ are the Van’t Hoff enthalpies for the first and second transitions, respectively, and *T*_m1_ and *T*_m2_ are the corresponding unfolding temperatures, defined as the temperature values halfway between states. In buffer, the transition temperatures produced by the fit are 59.80 ± 0.05 and 74.28 ± 0.02 °C, corresponding to the unfolding of the two sections. The *T*_m_ values indicate a high degree of structural stability, likely due to the addition of a non-native disulfide bond in the design of the ImmTAC molecules.^28^ The addition of sucrose had the effect of increasing the thermal stability, with a continuous increase in *T*_m_ values as sucrose concentration was increased; the highest concentration measured here (26.6% (w/v) sucrose) increased the unfolding temperatures by 3.48 and 4.82 °C, respectively (Figure 3b). The presence of NaCl (Figure 3a) had no effect on the structural stability of the protein, indicating that differences in Δ*H* between conditions with and without NaCl are not due to changes in structure. The highest NaCl concentration measured was 0.78% (w/v), which is comparable to the NaCl concentration in PBS buffer.

### Observation of Oligomeric States

**Figure 4:**
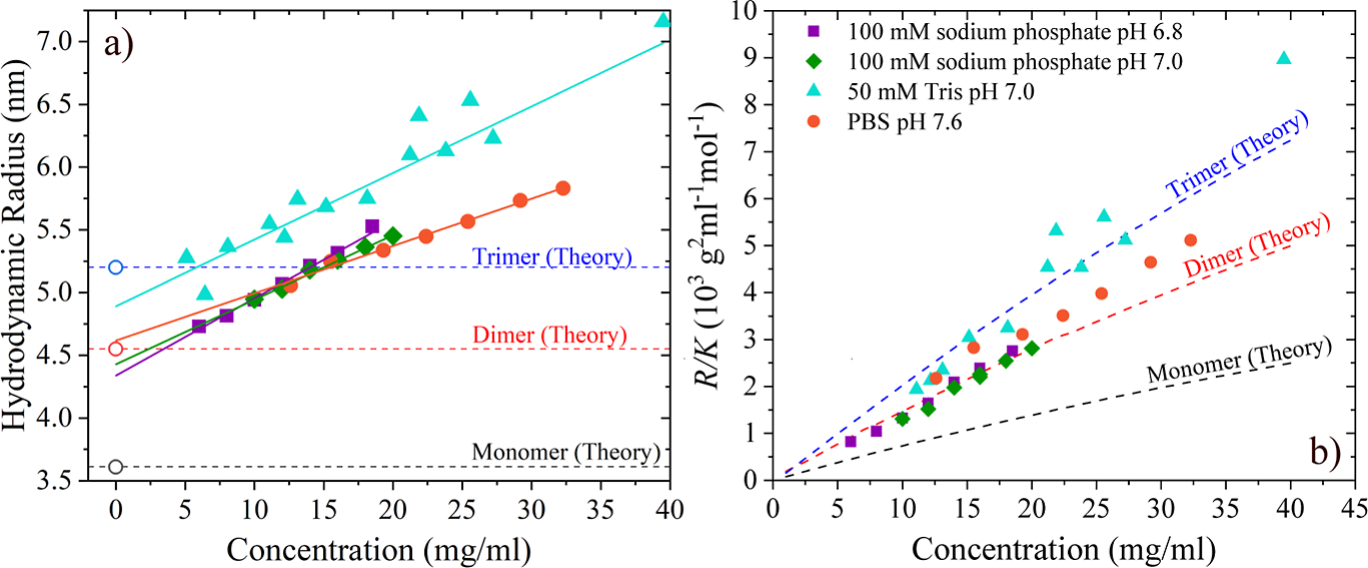
(a) Hydrodynamic radius of ImmTAC1 measured by DLS as a function of concentration. Solid lines are linear fits to the data. Open symbols represent predicted hydrodynamic radius values at infinite dilution (calculated using eq 17) for the monomer, dimer, and trimer, respectively. (b) Scattered light intensity as a function of concentration. Dashed lines are theoretical scattering intensities for the monomer, dimer, and trimer, respectively.

Using diffusion data obtained using DLS, hydrodynamic radius may be calculated using the Stokes–Einstein equation
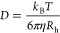
16where η is the viscosity of the solvent. Resulting radii are plotted in Figure 4a with a linear fit. Using eq 4 and again assuming that *R*_g_/*R*_h_ = 0.775, we estimate the theoretical hydrodynamic radius to be
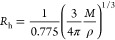
17

This relationship is in good agreement with experimental data reported by Smilgies and Folta-Stogniew,^29^ who compared protein molecular weights and hydrodynamic radius values obtained by DLS. Theoretical hydrodynamic radii for an ImmTAC1 monomer, dimer, and trimer are shown as dotted lines in Figure 4a. Measured values of *R*_h_ extrapolated to infinite dilution lie between theoretical values for the monomer and trimer, suggesting that there are some oligomers present even at the lowest concentrations. In Figure 4b, scattered light intensities measured by SLS are plotted along with theoretical scattering intensities for monomer, dimer, and trimer solutions, respectively, calculated from the scattering theory.^30^ These curves assume monodisperse solutions with monomer, dimer, and trimer molecular weights, respectively. In agreement with the hydrodynamic radii, the measured scattering intensities suggest that the average particle size is larger than a monomer but (mostly) smaller than a trimer. It is important to note that both of these techniques give an estimation of “average” size, for example, a measurement of *R*_h_ or scattered intensity corresponding to “trimer” could in fact result from a mixture of a monomer and a range of small oligomers. We also note that *B*_22_ and *k*_D_ by definition reflect pairwise interactions. Since we have determined that oligomers are present, these parameters reflect a combination of effects including any reversible oligomerization as well as nonspecific interactions (e.g., excluded volume effects) averaged over all possible combinations of oligomer types. As such, the reported values reflect “effective” parameters. Nonetheless, given the low polydispersity of the samples, the measured value reflects attractive interactions between molecules present in the solution.

**Figure 5:**
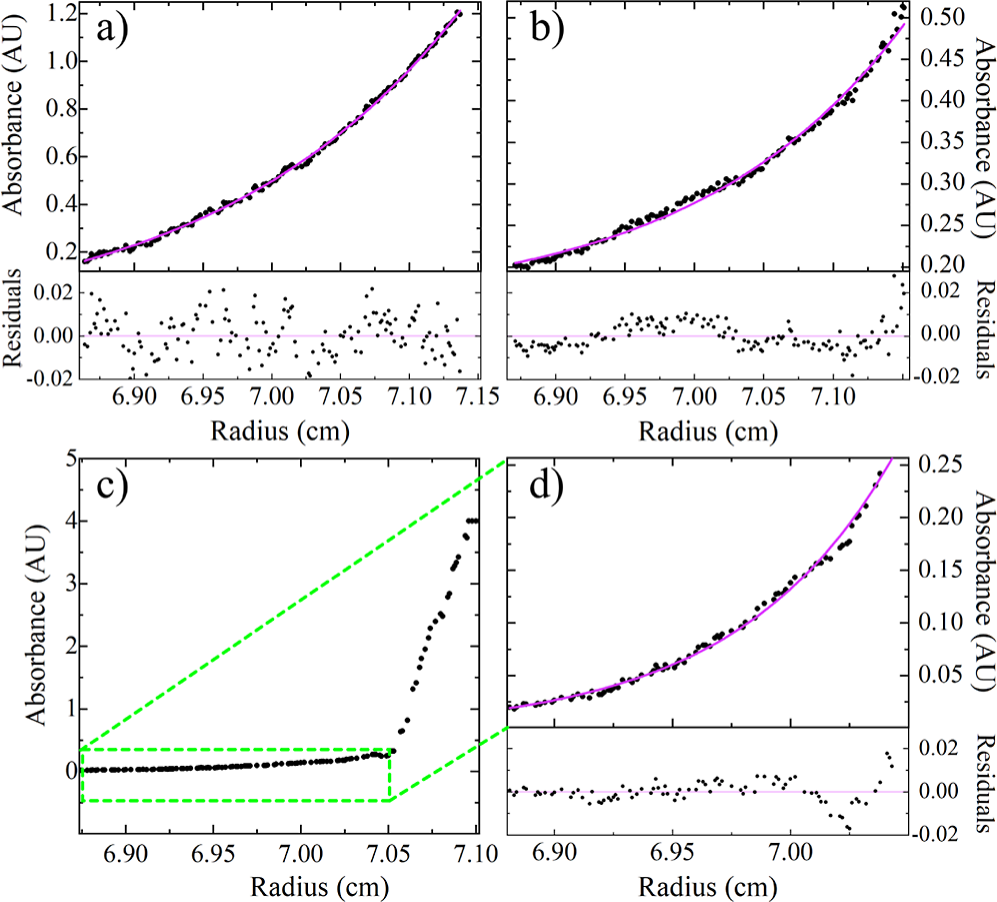
Sedimentation equilibrium AUC data for ImmTAC1 measured: (a) 1 mg/mL at 9000 rpm, (b) 11 mg/mL at 6000 rpm, and (c,d) 11 mg/mL at 15,000 rpm. Purple lines are fits to the data.

To determine the oligomer sizes consistent with the DLS and SLS data, we employed sedimentation equilibrium analytical ultracentrifugation (SE-AUC). Data were fitted using the SEDPHAT software^31^ to the equation for a single ideal noninteracting species^32^
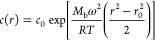
18where *c*_0_ is the concentration at a reference radius *r*_0_, ω is the rotor speed, and *R* is the gas constant. *M*_b_ = *M*_w_(1 – νρ_s_) is the buoyant mass of the particle, with ν the partial specific volume of the particle and ρ_s_ the solvent density. At 1 mg/mL (Figure 5a), the data show a good fit to a single ideal species with a fitted molecular weight of 78.00 kDa, in good agreement with the theoretical molecular weight for a monomer based on the amino acid sequence (77.2 kDa). At 11 mg/mL (Figure 5b), attempting to fit the data to a single ideal species results in a nonideal distribution of residuals, pointing to some polydispersity. The molecular weight from the fit was 209.70 kDa (2.72 times the theoretical monomer weight). When the 11 mg/mL sample was centrifuged at a higher speed of 15,000 rpm, the data showed two distinct sedimentation pools (Figure 5c). Analyzing the lower molecular weight species (Figure 5d), there is a good fit to a single species with a fitted molecular weight of 80.10 kDa. This suggests that a non-negligible amount of the monomer is present, along with a higher-order oligomer, with the total molecular weight pointing toward the trimer.

Given that our data indicate that net PPIs are dominated by electrostatic attraction, the formation of small oligomers is a reasonable conclusion. We may rule out partial unfolding or other structural changes as the cause of oligomerization since the unfolding temperatures and unfolding enthalpies that we measured (Figure 3 and Supporting Information) indicate a high degree of structural stability at room temperature, with the stability not significantly enhanced by the addition of common stabilizing excipients. It is unlikely that there are any structural changes occurring at the temperature (20 °C) at which the DLS and SLS were carried out. This further supports that the assembly into higher-order structures is due to the PPIs rather than any partial unfolding of the protein structure.

To confirm that this self-assembly is a feature of ImmTACs as a class of molecules, rather than ImmTAC1 alone, light scattering data was taken for a second ImmTAC molecule, ImmTAC2, which has an even more pronounced surface charge anisotropy. The structure is highly conserved across these two ImmTAC molecules; the RMSD of the two structures was found to be 0.386 Å when 500 similar atom pairs were matched in the sequence backbone for AlphaFold models of the two molecules. While structurally similar, ImmTAC1 and ImmTAC2 have different surface amino acids due to targeting different epitopes. As seen in Figure S13, this results in different surface charge distributions. ImmTAC2 has a pronounced difference between two of its domains (in contrast with ImmTAC1, the boundary between the charged regions occurs in the middle of the TCR domain). ImmTAC2 has a greater difference between its isoelectric points, with the two regions having almost equal and opposing net charges at physiological pH (see Figure S13). Data for ImmTAC2 in PBS was limited to samples below 12 mg/mL since concentrating to higher values resulted in visible precipitation. Even at lower concentrations, ImmTAC2 strongly aggregates under these solution conditions (aggregation was so apparent that it was not possible to measure a *B*_22_ or *k*_D_ value), showing that its tendency for self-association is much stronger than that demonstrated for ImmTAC1. This suggests that the greater degree of charge anisotropy and lower net charge lead to a more pronounced self-association behavior.

Comparing the data from the various solution conditions presented helps to shed light on patchy charge–charge attraction as the proposed mechanism for self-assembly. For example, for ImmTAC1 in PBS, which has an ionic strength of 167 mM, the net interactions are less attractive than those in 100 mM sodium phosphate buffer with no additional salt. The addition of salt in PBS screens the charge, counterintuitively making it more stable. In molecules where the charge distribution is more isotropic, increased screening by addition of salt would normally be associated with a destabilizing effect due to increase in attractive PPIs. This effect has been demonstrated extensively in the past literature for molecules without surface charge patchiness such as monoclonal antibodies (mAbs).^33,34^ Our data, which shows the opposite effect, is consistent with past studies where charge anisotropy leads to instability, which can be reduced by the addition of salts.^35,36^ Contrary to the isotropic case, charge–charge attraction of opposite polarity domains needs to be considered to explain this inverted relationship between stability and ionic strength.

While reports of anisotropic charge distributions leading to net-attractive PPIs for proteins are rare, more work has been explored for colloidal systems where “patchy” particles have been studied widely by coarse-grained simulations.^37−39^ Janus particles are particles with two distinct surfaces or regions with different properties. Charged Janus particles, colloids exhibiting positive charge on one side and negative charge on the other, have come to be known as inverse patchy particles (IPCs). This may be thought of as the simplest possible coarse-grained model of the surface charge distribution of ImmTAC1, which has a positively charged end and a negatively charged end (upper section of the molecule in Figure 1c). Simulations of IPCs have been shown produce multiple phases including crystals, gels, and clusters^40−42^ ranging from a trimer to higher-order multimers. At lower concentrations, size-limited clusters are formed, which supports the self-assembly observed here.

The question remains as to why the protein appears to form predominantly trimers (by AUC) and, on the time scales of our experiments, do not proceed to form higher-order oligomers. In all conditions measured, the polydispersity index (PDI) is smaller than 0.18 and, in all conditions other than Tris, is smaller than 0.07 (see the Supporting Information), indicating that no large aggregates are present. A species with at least double the hydrodynamic radius generally needs to be present in order to see the polydispersity reflected in an increased PDI value, which corresponds to an 8-fold increase in molecular weight. This indicates that the oligomers being formed are smaller than 8-mer. One possible explanation is the asymmetry between the sizes of the two charged regions. The TCR section which carries a charge of −15.9 at pH 7.0 (see Figure 1d) has a solvent accessible surface area (SASA) of 215.2 nm^2^, while the anti-CD3 section carries a charge of 3.8 at the same pH with a SASA of 121.2 nm^2^ (SASA values were calculated using the “measure sasa” tool available in UCSF ChimeraX^43^). It has been shown in simulations of both spherical and circular IPCs that as the difference in size between the two regions increases, the number of attractive contacts that each particle can make without forming repulsive contacts decreases, leading to lower coordination number and limiting cluster sizes.^40,42^ Another possible explanation is that once a small oligomer is formed, the total charge is sufficient to stabilize the oligomer. Studies have reported that increasing the charge of a protein, either through mutagenesis^44−46^ or nonspecific binding of small charged molecules to the surface,^47,48^ results in an increase of the colloidal stability of the molecule in solution by increasing the intermolecular electrostatic repulsion at low ionic strength. Hence, we hypothesize that upon small oligomer formation, the local charge anisotropy is reduced, producing an oligomer with a more balanced distribution of charge across the surface and a more isotropic net interaction potential. This explanation is consistent with the higher-order aggregation observed for ImmTAC2; as seen in Figure S13, ImmTAC2 has a net charge close to zero for the solution conditions studied here. Therefore, the formation of small oligomers would not result in a highly charged species as it would for ImmTAC1, leaving open the possibility of higher-order oligomer formation.

It is interesting to note that during preparation of all samples (for both light scattering and AUC experiments), the highest concentration samples were prepared first and diluted to obtain lower concentrations. During AUC, the oligomers observed in the 11 mg/mL sample were not observed in the 1 mg/mL sample, indicating that the higher-order structures had dissociated in this diluted sample. However, the hydrodynamic radii measured by DLS close to 1 mg/mL indicate the presence of species larger than the monomer, implying that dilution alone may not be enough to cause the oligomers to dissociate. Samples close to 1 mg/mL may also undergo higher-order aggregation as demonstrated by an elevated polydispersity index and hydrodynamic radii (see the Supporting Information). Due to this, it is not necessarily possible to state whether oligomers are present at the lowest concentrations. Indeed, the fact that centrifugation of the 11 mg/mL sample at higher speeds was able to remove the oligomeric species altogether leaving the monomer behind suggests that there is no association/dissociation equilibrium between monomers and small oligomers, at least on shorter time scales. It is possible that the formation of a trimer is weakly reversible and that shear forces from the ultracentrifugation itself are enough to cause dissociation at low concentration.

The self-assembly demonstrated here represents a challenge for the formulation of ImmTAC molecules, which is likely to be present in many other types of engineered fusion proteins. It is clear from decades of previous work that self-assembly is often detrimental to the efficacy and safety of biopharmaceutical proteins since this can both hinder active sites and increase immunogenicity,^49,50^ posing a problem for administering them as a pharmaceutical product. Since all ImmTACs are bispecific fusion proteins, this challenge will exist to varying extents for the entire class of molecules, evidenced by the fact that it presents itself in both ImmTAC1 and ImmTAC2 to varying degrees based on the extent of charge anisotropy. Indeed, many promising biopharmaceutical molecules being developed in recent years are bispecific fusion proteins, making this a pressing issue that one needs to be aware of when developing new biologics. The implications for formulation of these molecules is that measures need to be taken to mitigate possible instability brought about when the surface characteristics of the protein lead to significant anisotropy in noncovalent, weak protein–protein interactions. As evidenced in this study, selecting the optimum buffer conditions is of the utmost importance. Other excipients may be utilized, for example, surfactants such as polysorbates, which adsorb to the surface of proteins to create protein–surfactant complexes, creating a steric barrier and reducing the likelihood of charge–charge patch interactions, may be an attractive candidate. Indeed, adding 0.02% (w/v) Tween 80 reduced the PDI when added to ImmTAC2 in 100 mM sodium phosphate at pH 7.4 (see the Supporting Information).

## Conclusions

We have shown that ImmTACs exhibit an anisotropic distribution of surface charge akin to a Janus particle due to its design as a fusion of two proteins with different isoelectric points. Due to this feature, the molecule exhibits net attractive interactions, dominated by electrostatic interactions between regions of unlike charges on different molecules. This in turn leads to the self-assembly of small oligomers, which coexist with monomers. By comparing two different ImmTACs with varying degrees of Janus-like charge anisotropy, we have concluded that this is a feature that is likely to be present in other ImmTACs due to their designed nature as synthetic molecules. The degree of colloidal instability scales with the severity of Janus-like nature.

This work has implications for the rational design of protein and/or peptide self-assembly; it may be feasible to direct the desired self-assembled states by engineering molecules with different configurations of anisotropic surface charge.

Understanding the impact of protein structure and anisotropy on PPIs and self-assembly is also important for formulation; ultimately, these proteins are designed to be therapeutic molecules and as such require stable solution conditions in order to be used for treatment. Understanding the solution behavior of anisotropic molecules as Janus particles opens the door for more rational approaches to formulation and selection of formulation excipients in the future. This has implications not only for ImmTACs but also for the stability of all bispecific fusion proteins, an important and emerging category of biologics.

## Methods and Materials

### Sample Preparation

ImmTAC molecules were provided by Immunocore as 2.4 mg/mL solutions in PBS. Reagents were purchased from Thermo Fisher Scientific and Merck-Millipore. Buffers containing 100 mM sodium phosphate at pH 6.8 and 7, 50 mM Tris at pH 7.0, and PBS at pH 7.6 were all prepared in Milli-Q water (system by Merck Millipore) by dissolving the appropriate amount of salts. All buffers contained 0.02% w/v sodium azide to prevent microbial growth and were degassed and filtered using a 0.45 μm, 47 mm cellulose filter (Millex or Sartorius). Amicon Ultra-4 (10 kDa molecular weight cutoff) from Merck-Millipore were used to concentrate protein solutions and buffer exchange to the appropriate buffer. Protein concentrations were measured by UV spectroscopy at a wavelength of 280 nm.

### Light Scattering

SLS and DLS were performed using an ALV/CGS-3 goniometer and ALV/LSE-5004 Multiple Tau Digital correlator with a 638.2 nm HeNe laser. The sample environment was kept at 20 °C using a Thermo Scientific DC30-K20 water bath, and measurements were taken at a 90° scattering angle. Protein samples were centrifuged at 13,000*g* for 1 h prior to measurements in order to remove aggregates. Measurements were taken starting with the highest concentration and performing a serial dilution to obtain the lower concentrations.

### Analytical Ultracentrifugation

Sedimentation equilibrium analytical ultracentrifugation (SE-AUC) was performed using a Beckman XL-I analytical ultracentrifuge with dual-sector Epon-filled centerpieces with quartz glass in an AN-50 Ti rotor. Samples were sedimented at 1907, 4292, and 11,924*g* at concentrations of 1 and 11 mg/mL.

### Nano Differential Scanning Fluorimetry

Nano-DSF was carried out using an Applied Photophysics SUPR-DSF. 25 μL portion of the sample at 0.5 mg/mL was pipetted into 384-well plates. Temperature ramps were performed between 20 and 100 °C at a rate of 1 °C per minute. A high-power 280 nm LED was used for excitation, and the resulting fluorescence spectra were measured for wavelengths between 310 and 420 nm with a 25 ms integration time. The ratio of fluorescence intensities at 355 and 330 nm was calculated at each temperature.

## Supporting Information Available

Plots of thermal unfolding, unfolding temperatures, and unfolding enthalpies measured by nano-DSF under various excipient conditions; plots of *R*/*Kc*, hydrodynamic radii, and polydispersity indices of ImmTAC1 measured by DLS; comparison between AlphaFold and experimentally determined structures of ImmTAC1; comparison between ImmTAC1 and ImmTAC2; DLS data for ImmTAC2 with and without Tween 80; and fitting for polydispersity index from DLS (PDF)
