# Correction to “Synthetic T‑Cell Receptor-like Protein Behaves as a Janus Particle in Solution”

**DOI:** 10.1021/jacs.6c00896

**Published:** 2026-02-09

**Authors:** Emily Sakamoto-Rablah, Jordan Bye, Arghya Modak, Andrew Hooker, Shahid Uddin, Jennifer J. McManus

In Figure 2­(a) of the published article, the units on the *y*-axis were incorrectly given as “mol g^–1^”. The correct units should be “μmol g^–1^”. The complete, corrected Figure 2 is shown below. This correction does not affect the data, analysis or conclusions of the paper.

**Figure 2:**
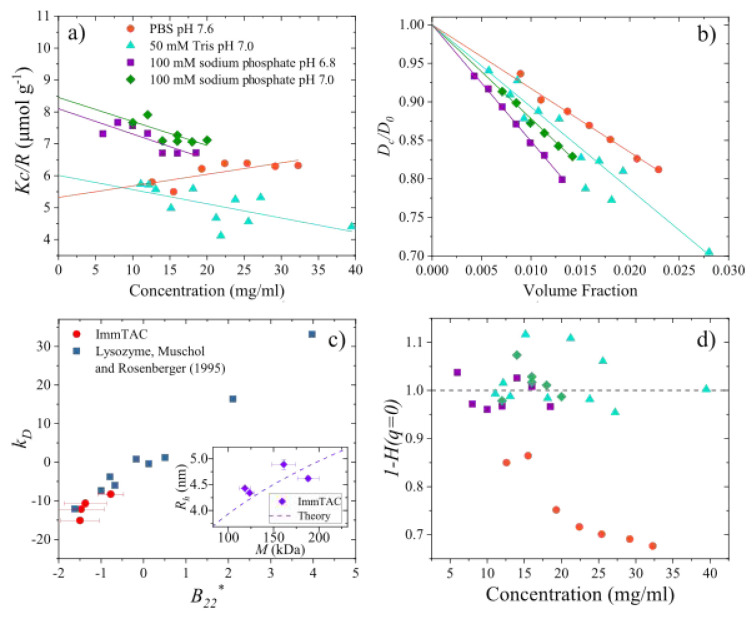
SLS data plotted as a Debye plot. Straight lines are linear fits to data with slopes equal to the second virial coefficient *B*_22_. (b) Diffusion data measured by dynamic light scattering (DLS). Straight lines show fits to the data with the slope equal to interaction parameter *k*_D_. (c) Relationship between the interaction parameter and second virial coefficient for ImmTAC1 and lysozyme. Data for lysozyme is reproduced from Muschol and Rosenberger.^15^ Inset shows the relationship between the measured hydrodynamic radius and molecular weight for ImmTAC1 along with the expected relationship (dashed line) calculated from the theory (eq 17). (d) Hydrodynamic function calculated using eq 12 as a function of concentration.

