# Supplementary material for: Synthetic T-Cell Receptor-like Protein Behaves as a Janus Particle in Solution
Source: J Am Chem Soc. 2024 Dec 19;147(1):247–56. doi: 10.1021/jacs.4c08932 (PMC11726545; doi:10.1021/jacs.4c08932)
Supplement: Supplementary file 1 — ja4c08932_si_001.pdf [file ja4c08932_si_001.pdf]

Supporting Information for: "A synthetic T-cell receptor-like protein behaves as a Janus particle in solution"

Emily Sakamoto-Rablah,[†] Jordan Bye,[‡] Arghya Modak,[‡] Andrew Hooker,[‡] Shahid Uddin,[‡] and Jennifer J McManus[*,†,¶]

†*HH Wills Physics Laboratory, University of Bristol, BS8 1TL, United Kingdom*

‡*Immunocore Limited, 92 Milton Park, Abingdon OX14 4RY, United Kingdom*

¶*Bristol Biodesign Institute, University of Bristol, BS8 1QU, United Kingdom*

E-mail: jennifer.mcmanus@bristol.ac.uk

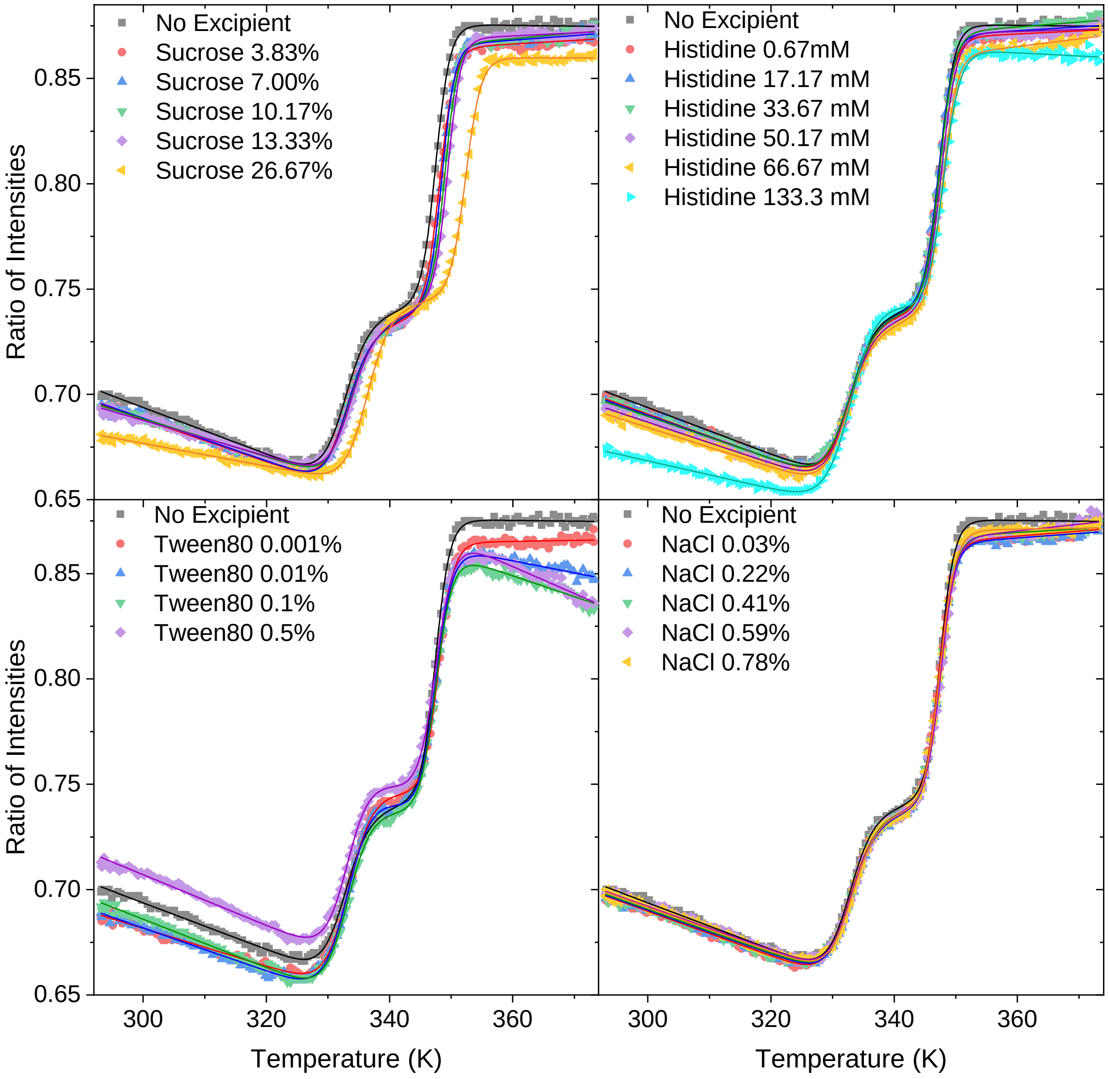

Figure S1: Thermal unfolding curves for ImmTAC1 in the presence of various concentrations of sucrose, histidine, tween and NaCl. Percentages are (w/v)%. Lines are fits to the data using Equation 13.

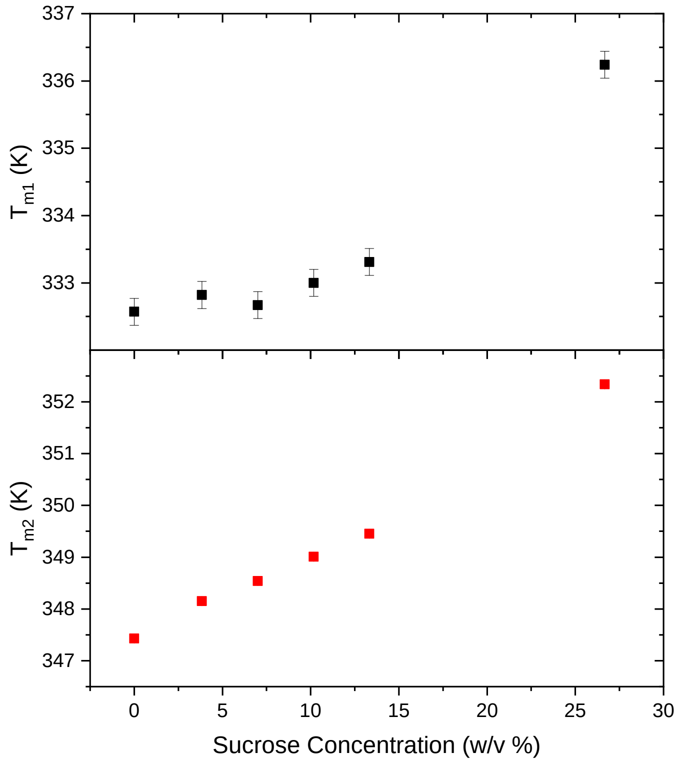

Figure S2: Unfolding temperatures for ImmTAC1 in the prescence of various concentrations of sucrose. The error bars represent the standard errors obtained from the fit. Error bars are present on all points but in some cases smaller than the data points.

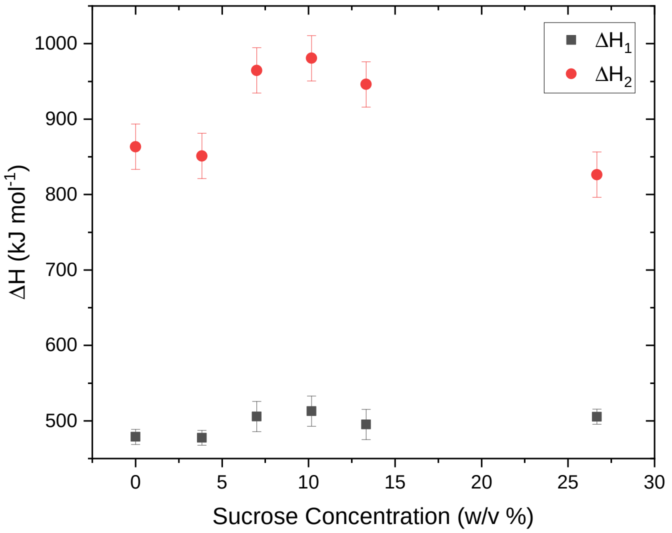

Figure S3: Unfolding ethalpies for ImmTAC1 in the prescence of various concentrations of sucrose. The error bars represent the standard errors obtained from the fit.

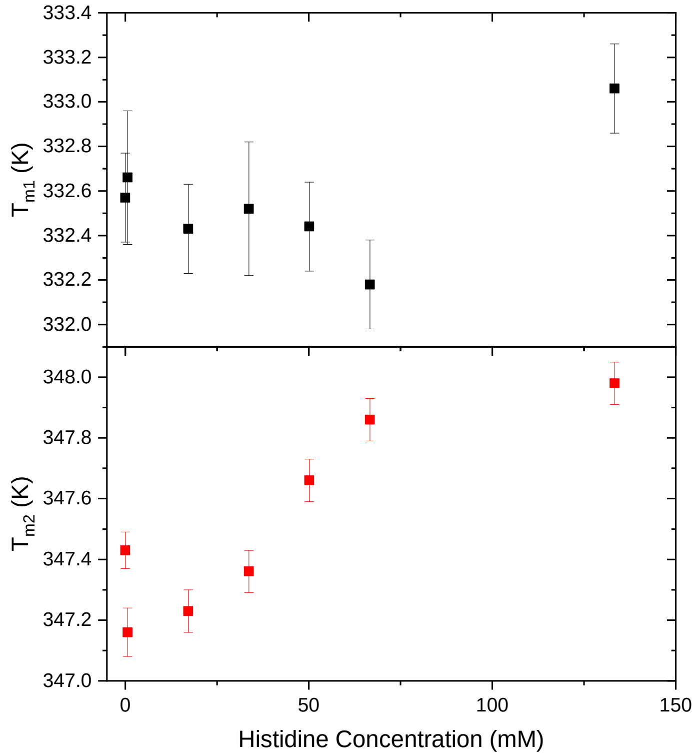

Figure S4: Unfolding temperatures for ImmTAC1 in the prescence of various concentrations of histidine. The error bars represent the standard errors obtained from the fit.

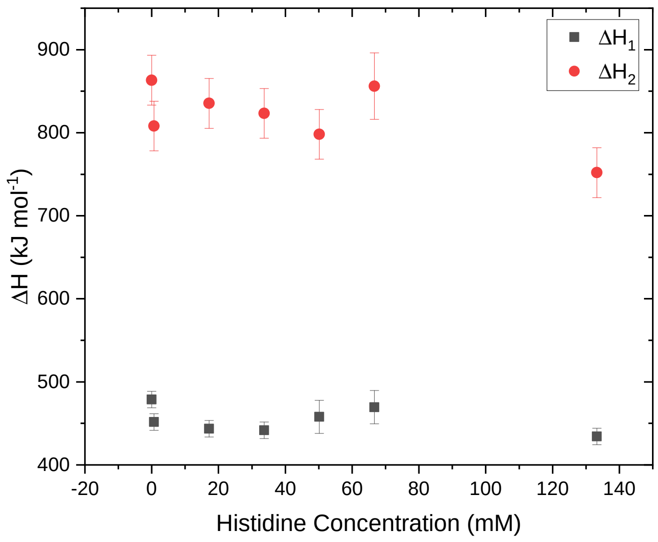

Figure S5: Unfolding enthalpies for ImmTAC1 in the prescence of various concentrations of histidine. The error bars represent the standard errors obtained from the fit.

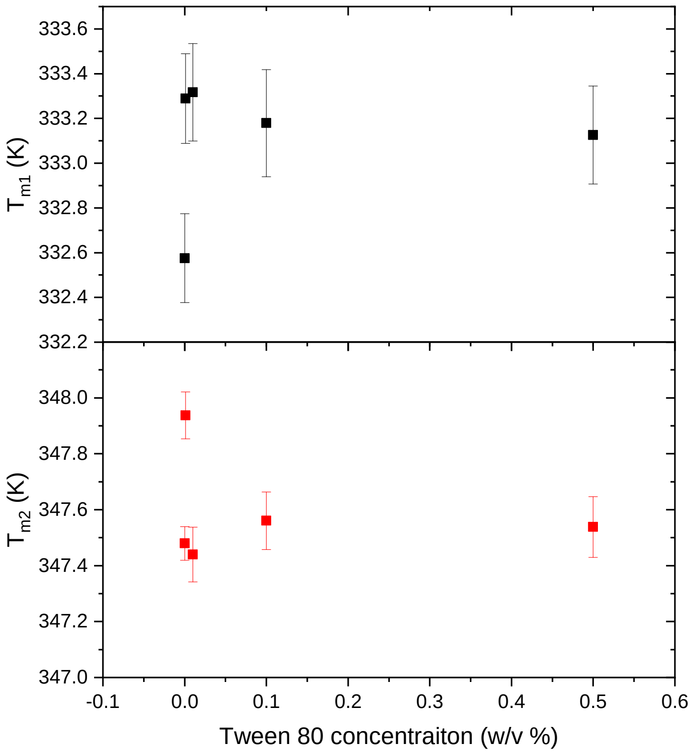

Figure S6: Unfolding temperatures for ImmTAC1 in the prescence of various concentrations of Tween 80. The error bars represent the standard errors obtained from the fit.

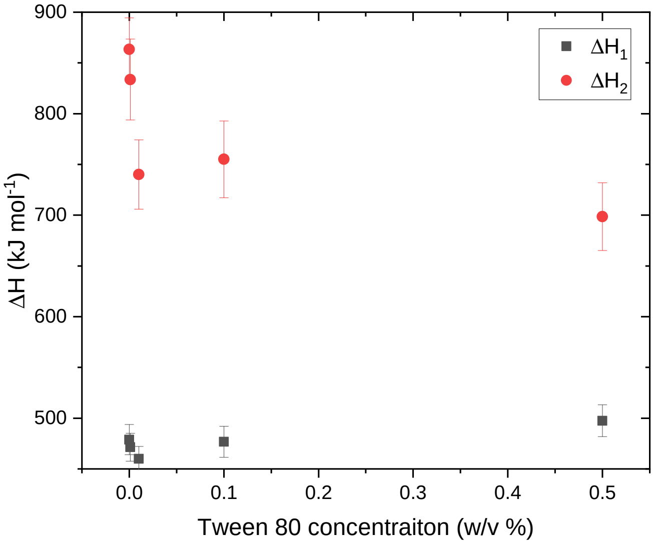

Figure S7: Unfolding enthalpies for ImmTAC1 in the prescence of various concentrations of Tween 80. The error bars represent the standard errors obtained from the fit.

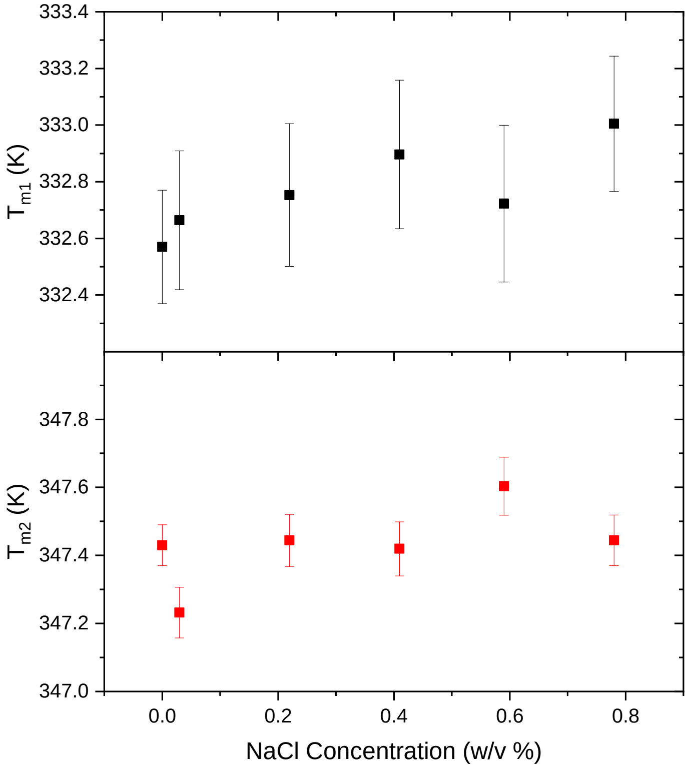

Figure S8: Unfolding temperatures for ImmTAC1 in the prescence of various concentrations of NaCl. The error bars represent the standard errors obtained from the fit.

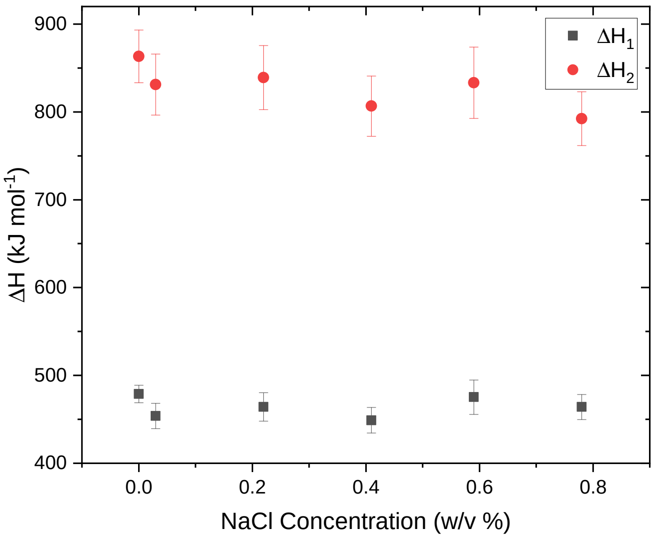

Figure S9: Unfolding enthalpies for ImmTAC1 in the prescence of various concentrations of NaCl. The error bars represent the standard errors obtained from the fit.

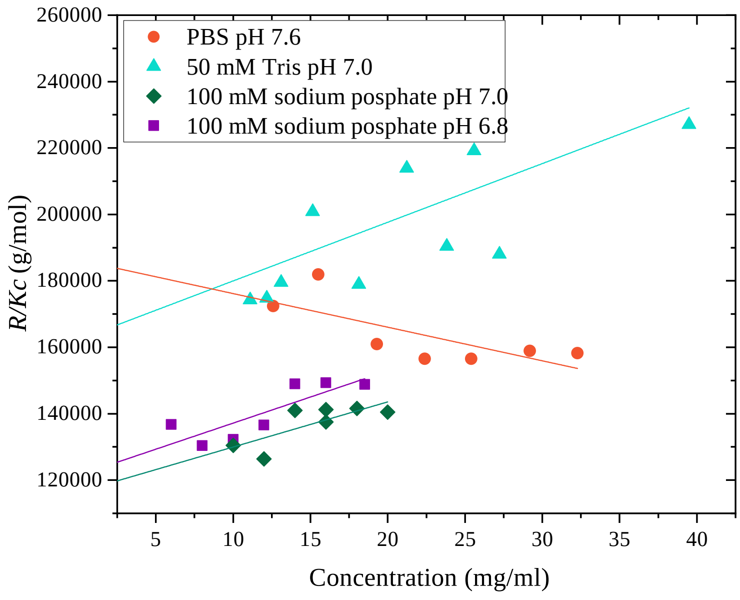

Figure S10: Plots of R/Kc for ImmTAC1 measured by SLS in various buffer conditions. Straight lines are linear fits to the data.

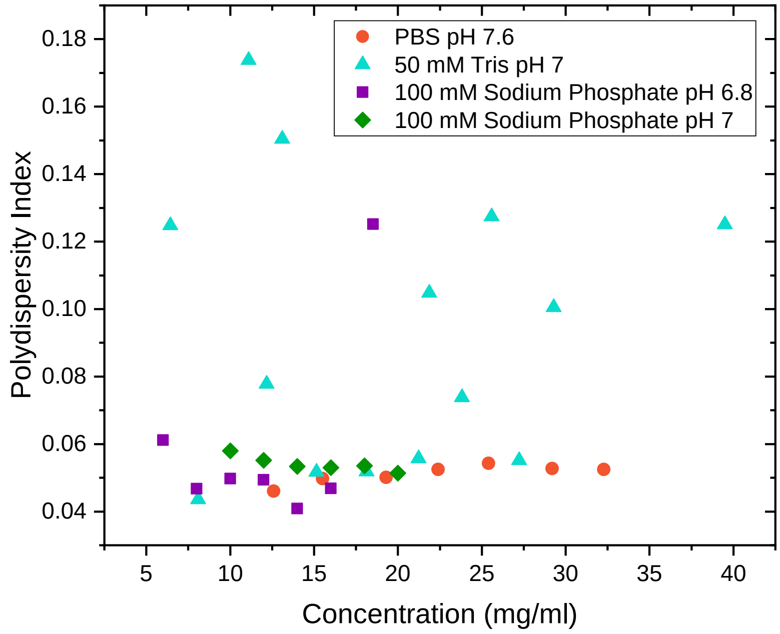

Figure S11: Polydispersity index values for ImmTAC1 measured by DLS in various buffer conditions, calculated using Equation S2.

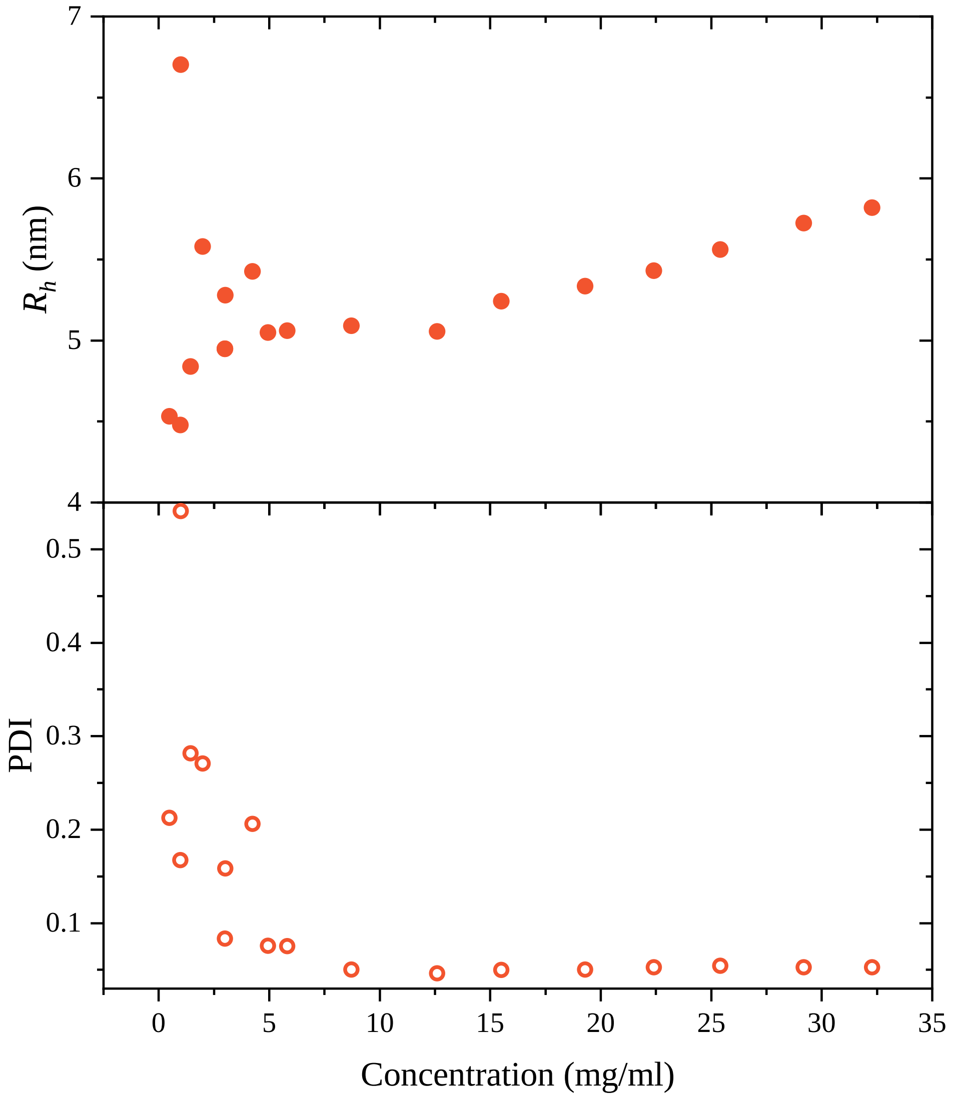

Figure S12: Hydrodynamic radii and polydispersity indices for ImmTAC1 measured by DLS in pH 7.6 PBS.

Intensity correlation functions measured by dynamic light scattering were fitted to the second order polynomial

$$ln(g_2(\tau)) = -2q^2 D_c \tau + q^4 \tau^2 (\delta D_c)^2, \tag{S1}$$

The polydispersity index (PDI) is then defined as

$$PDI = \frac{(\delta D_c)^2}{D_c^2} \tag{S2}$$

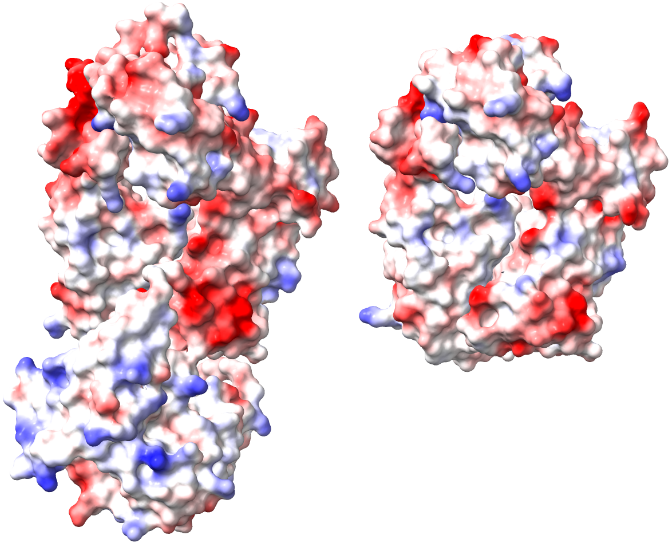

Figure S13: Left: Surface charge distribution for Alphafold predicted structure of ImmTAC1. Right: surface charge distribution for T-cell receptor, PDB:2P5E.

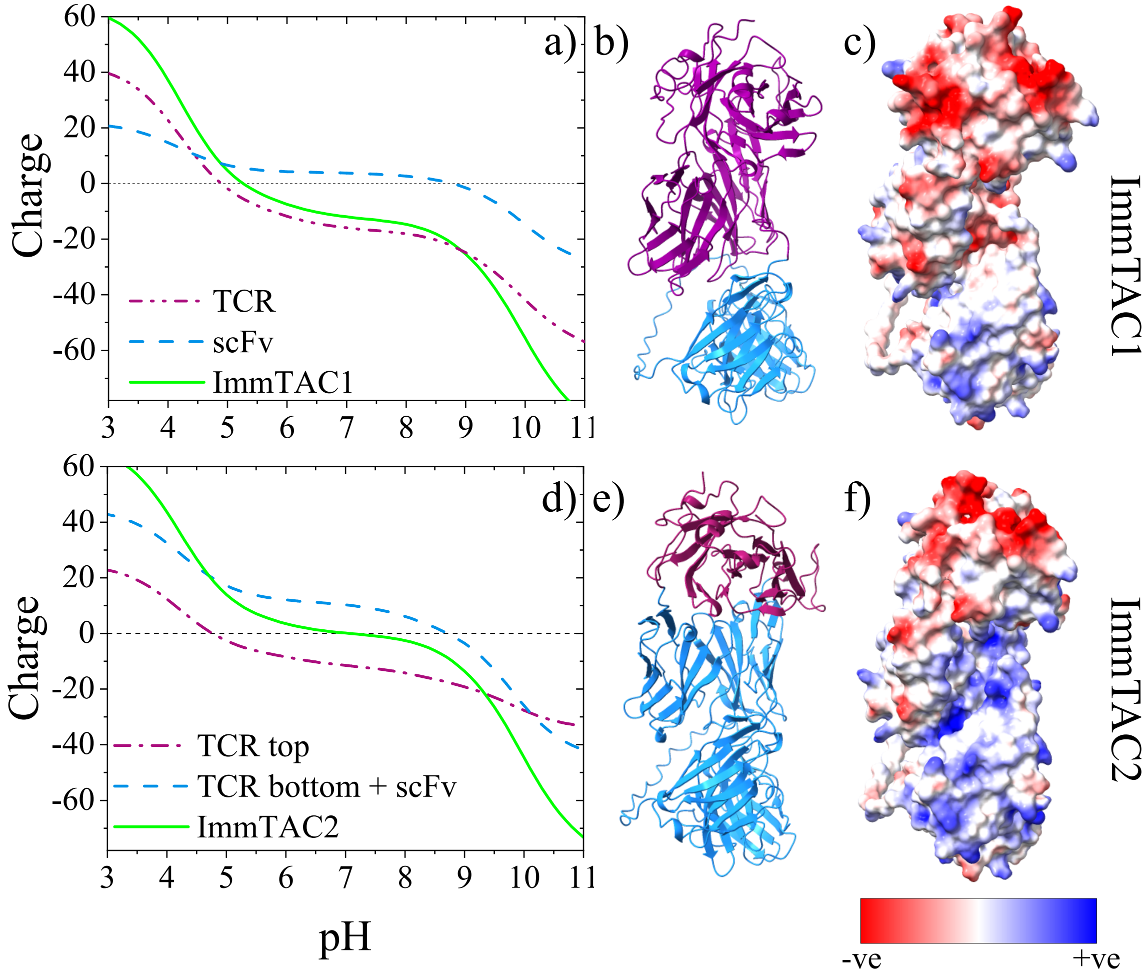

Figure S14: a) Predicted net charge of ImmTAC1 and its constituent domains. b) Alphafold structural prediction for ImmTAC1. The colours show the domains for which net charge has been calculated - the TCR and anti-CD3 scFv domains. c) Surface charge distribution of ImmTAC1. d) Predicted net charge of ImmTAC2 and its constituent domains. e)Alphafold structural prediction for ImmTAC2. The colours show the domains for which net charge has been calculated - the top section of the TCR, and the bottom section of the TCR combine with the anti-CD3 scFv. f) Surface charge distribution of ImmTAC2.

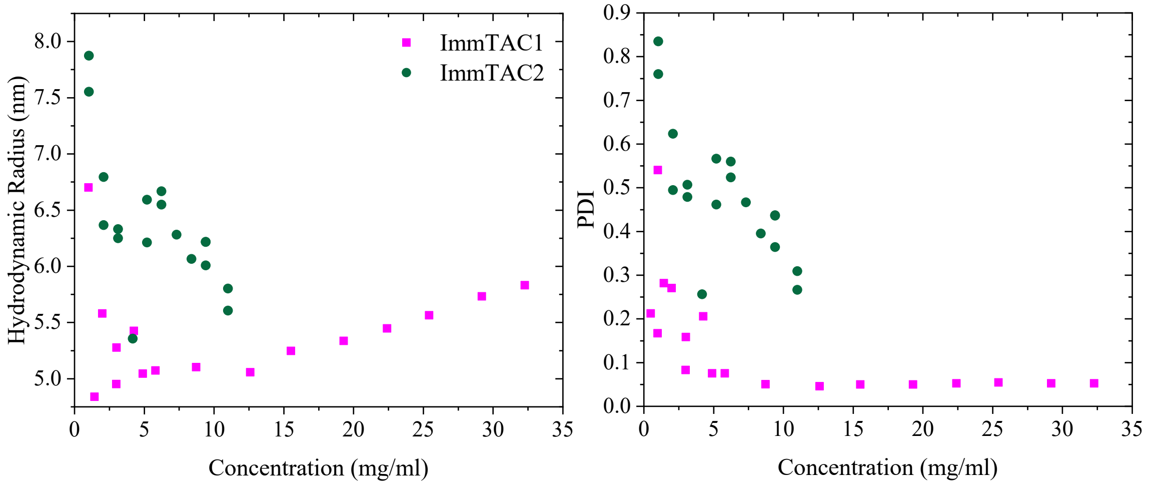

Figure S15: a) Hydrodynamic radii and b) polydispersity indeces of ImmTAC1 and ImmTAC2 measured at various concentrations by DLS in PBS at pH 7.6.

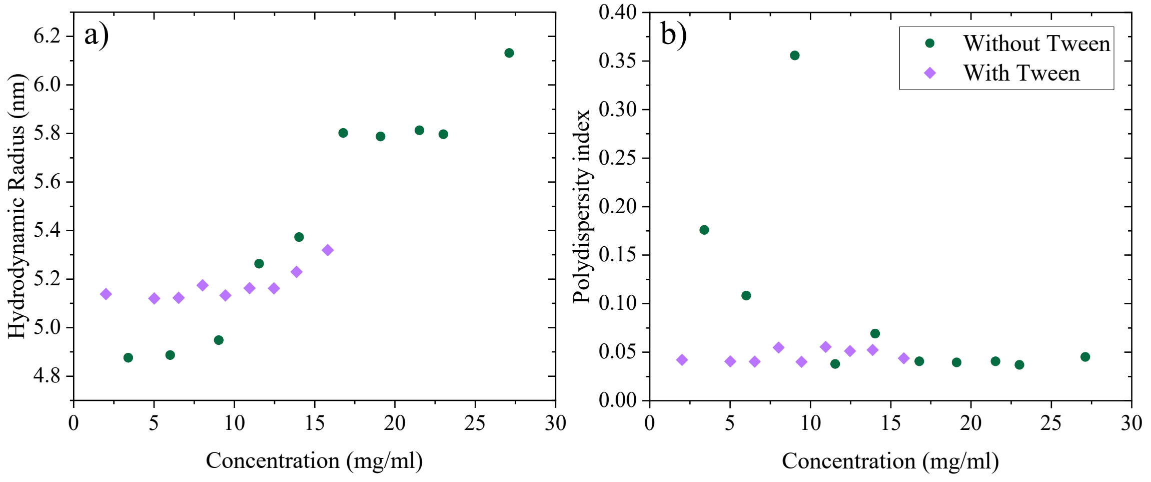

Figure S16: a) Hydrodynamic radii and b) polydispersity indices of ImmTAC2 in 100 mM phosphate with and without the addition of 0.02 % (w/v) Tween 80.
